# Cardiac cryptographers: cracking the code of the epitranscriptome

**DOI:** 10.1093/eurheartj/ehae057

**Published:** 2024-02-10

**Authors:** Charles P Rabolli, Federica Accornero

**Affiliations:** Department of Biomedical Engineering, The Ohio State University, Columbus, OH 43210, USA; Department of Molecular Biology, Cell Biology and Biochemistry, Brown University, Sidney E Frank Hall for Life Sciences, 185 Meeting St, Providence, RI 02912, USA; Department of Molecular Biology, Cell Biology and Biochemistry, Brown University, Sidney E Frank Hall for Life Sciences, 185 Meeting St, Providence, RI 02912, USA

Independent from the nucleotide sequence dictated by DNA, native RNA is full of information, coated with over 170 chemical modifications (collectively known as the epitranscriptome) that modulate its stability, localization, translation efficiency, and many more facets of RNA processing, including even the codon meaning.^1^ Oftentimes, RNA modifications can be overlooked in part because of the difficulty in detecting their presence and/or studying their function. For a majority of modifications, especially in the cardiac system, their roles are completely unknown; a particularly egregious gap in knowledge when one considers that over 100 ‘RNA Modopathies’ (RNA modification disease-driving defects) have been described in humans,^2^ with potentially many more to come. This gap, however, should not be a cause for hesitation, but motivation. Much like the 2023 Nobel Prize in Physiology or Medicine was awarded for breakthrough discoveries on RNA Modifications, cardiac researchers too can make great strides by beginning to crack the epitranscriptome.

## The old and the new

In 1977, development of northern blotting allowed researchers to, for the first time, quantitatively measure RNA abundance. Less than a decade later, polymerase chain reaction (PCR) became the easiest and most ubiquitous way of studying RNA. PCR would be expanded upon during the mid-2000s with the advent of next-generation sequencing, a technology that would be used for RNA-Sequencing. These techniques have been the backbone of quantitative RNA biology, but more recent technological and computational advancements have enabled sequencing at the single-cell level and with spatial resolution. However, with the preponderance of available RNA datasets, why do we still so often fail to predict protein level changes from RNA, especially during dynamic processes like responses to stress or cell division^3^? Or, to paraphrase the namesake paradox of Nobel Laureate Enrico Fermi—where are the proteins?

Nowadays, global investigation of RNA commonly entails next-generation sequencing of RNA, a veritable misnomer given that it involves making complementary DNA (cDNA) copies from RNA that are in turn sequenced. Problematically, this greatly ignores the modification status, obfuscating full epitranscriptomic insight. To combat this shortcoming, there are two sequencing methods which directly yield information on RNA modifications. The first, Nanopore sequencing, passes single RNA molecules through membranous pores and decodes nucleotide and modification status through changes in electrical current as the nucleotides pass through the pores. These deflections in currents represent unique nucleotide signatures that, when compared computationally to known standards, allow for ‘calling’ of modified nucleotides. The main limitations of Nanopore sequencing are its error rate and the scarcity of identifiable modifications. Of the 170 chemical modifications, Nanopore can only detect a handful of these; however, its high throughput capability and its ability to map modifications in sequence context and at single molecule resolution makes it a promising technique for the modifications that it can detect.

The other method for direct investigation of the epitranscriptome is RNA mass spectrometry. liquid chromatography followed by mass spectrometry (LC-MS/MS) is the gold standard for modification analysis, as it can detect and precisely establish the chemical structure of all modifications. However, mass spectrometry requires a large amount of input RNA, which poses challenges for low-abundance transcripts and modifications. Furthermore, the fragmentation steps limit the length of the RNA transcripts that can be read, complicating the ability to relate detected modifications to single-transcript resolution.

## The heart of the matter

Studies of RNA modifications in the heart have predominantly focused on mRNA methylation. Nucleotide methylation is the most prevalent epitranscriptomic mark on RNA^4^ and is found to increase in ischaemic and non-ischaemic cardiomyopathy human heart samples, and porcine and murine models of myocardial infarction.^5^ N^6^-methyladenosine (m^6^A) is the single best characterized RNA modification in the heart with numerous studies highlighting its role on coding and non-coding RNAs in cardiac development and remodelling.^6^ Other prominent modifications, such as 7-methylguanosine (m^7^G), 5-methylcytosine (m^5^C), N^1^-methyladenosine (m^1^A), N^6^-2′-O-dimethyladenosine (m^6^A_m_), 2′-O-methylation (2′-OMe), and Pseudouridine (Ψ), have been scarcely studied in the heart, but represent potential areas of interest, given their established importance in other systems. Similarly, deamination of adenosine to inosine (A-to-I editing), catalysed by Adenosine Deaminase Acting on RNA (ADAR) enzymes, is increased in patients with congenital heart defects,^7^ but the reasons for this are not exactly clear. Taken together, when considering the established significance of the epitranscriptome^8^ and the combinatorial diversity of modifications,^9^ we should be excited about the potential therapeutic avenues that are currently uninvestigated but could prove pivotal in the future.

No doubt that studying the epitranscriptome will uncover a treasure trove of information still lurking in the heart. While published work addresses some questions regarding m^6^A specifically, overall, we are left with more questions than answers—if this one modification on RNA is so crucial, what kind of information could we discover from the remaining 170+ modifications? Does a specific modification function differently on different species of RNA? How does the location of the modification (i.e. coding sequence vs. UTR vs. non-coding RNAs) affect transcript and subsequent protein synthesis and/or gene expression? How do multiple modifications on the same RNA interact with one another?

## The road less travelled

**Figure 1:**
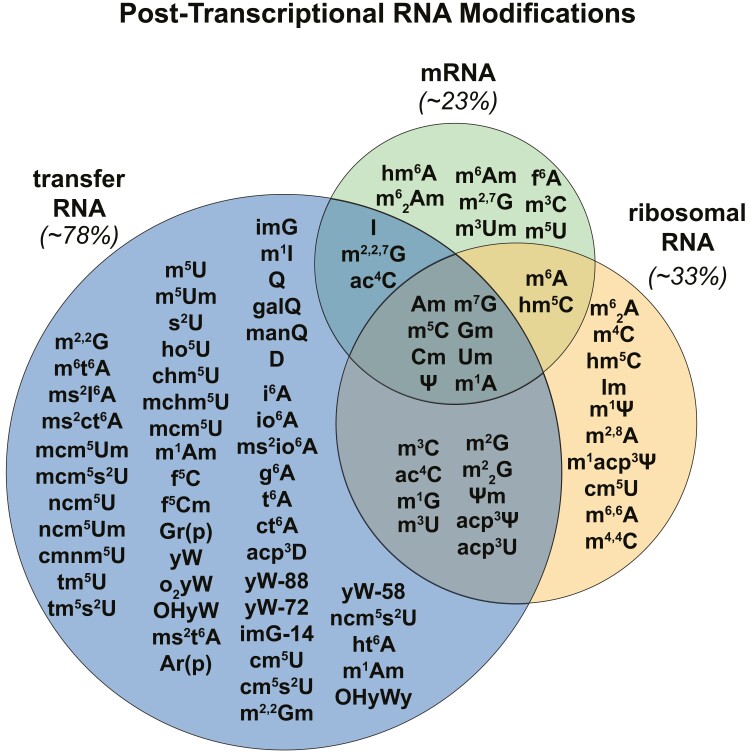
Representative eukaryotic RNA modification list based on current information revealing the dominance of chemical marks on non-coding RNAs vs. mRNA. While we are only showing Eukarya, the trends depicted here hold true for all domains of life. 2′-OMe is represented independently for each nucleotide as ‘Am’, ‘Cm’, ‘Gm’, and ‘Um’. Percentages represent the per cent of total modifications shown in the figure which are found on that species

When contemplating the rich landscape of the epitranscriptome, these questions become larger than the cardiac system alone and are being discussed at the highest levels of academic partnerships,^10^ but the unique features of the mammalian heart position researchers to address unanswered questions. In a post-mitotic cell, like a cardiomyocyte, there exists an exceptionally robust level of precision and control of post-transcriptional modifications, given the necessity for survival and minimal turnover capability. Yet, we still have not addressed the major lingering question—where are the proteins? Perhaps we in this field have been too myopic in our thinking. Majority of the epitranscriptomic studies in the heart focuses on mRNA, despite it bearing only a handful of the 170 known modifications. As René Descartes would likely tell us, it is time to remove our preconceived notions and to re-evaluate whether an inflated emphasis on mRNA is still justified. mRNA is neither the most prevalent RNA species, nor the most heavily modified (*Figure 1*). Bayesian probability would suggest a greater likelihood of discovering key regulatory modifications on RNA which are more diversely modified, like tRNA and rRNA. Inevitably, regardless of the RNA species, there is substantial information remaining to be uncovered. Deciphering the codes hidden in the epitranscriptome could usher in breakthroughs not only in basic and translational cardiovascular work, but in the molecular biology and drug therapeutic fields as a whole.

## Declarations

### Disclosure of Interest

All authors declare no confict of interest and disclosures are provided.
